# Intersectional effects of racial and gender discrimination on cardiovascular health vary among black and white women and men in the CARDIA study

**DOI:** 10.1016/j.ssmph.2019.100446

**Published:** 2019-07-04

**Authors:** G.S. Bey, B. Jesdale, S. Forrester, S.D. Person, C. Kiefe

**Affiliations:** a Department of Epidemiology, Gillings School of Global Public Health, University of North Carolina at Chapel Hill, 135 Dauer Dr. Chapel Hill, NC, 27599, USA; b Department of Population and Quantitative Health Sciences, University of Massachusetts Medical School, 55 Lake Ave North, Worcester, MA, 01655, USA

**Keywords:** USA, Identity pathology, Gendered race, Cardiovascular health, Intersectionality, Discrimination, Health inequities

## Abstract

Testing hypotheses from the emerging Identity Pathology (IP) framework, we assessed race-gender differences in the effects of reporting experiences of racial and gender discrimination simultaneously compared with racial or gender discrimination alone, or no discrimination, on future cardiovascular health (CVH). Data were from a sample of 3758 black or white adults in CARDIA, a community-based cohort recruited in Birmingham, AL; Chicago, IL; Minneapolis, MN, and Oakland, CA in 1985–6 (year 0). Racial and gender discrimination were assessed using the Experiences of Discrimination scale. CVH was evaluated using a 12-point composite outcome modified from the Life's Simple 7, with higher scores indicating better health. Multivariable linear regressions were used to evaluate the associations between different perceptions of discrimination and CVH scores two decades later by race and gender simultaneously. Reporting racial and gender discrimination in ≥2 settings were 48% of black women, 42% of black men, 10% of white women, and 5% of white men. Year 30 CVH scores (mean, SD) were 7.9(1.4), 8.1(1.6), 8.8(1.6), and 8.7(1.3), respectively. Compared with those of their race-gender groups reporting no discrimination, white women reporting only gender-based discrimination saw an adjusted score difference of +0.3 (95% CI: 0.0,0.6), whereas white men reporting only racial discrimination had on average a 0.4 (95% CI: 0.1,0.8) higher score, and scores among white men reporting both racial and gender discrimination were on average 0.6 (95% CI: 1.1,-0.1) lower than those of their group reporting no discrimination. Consistent with predictions of the IP model, the associations of reported racial and gender discrimination with future CVH were different for different racially-defined gender groups. More research is needed to understand why reported racial and gender discrimination might better predict deterioration in CVH for whites than blacks, and what additional factors associated with gender and race contribute variability to CVH among these groups.

## Highlights

• We examined differences among black and white women and men in the impact of racial or gender discrimination, or both, on cardiovascular health.
• White men appear particularly susceptible to poorer cardiovascular health associated with racial and gender discrimination.
• Dominant status groups may be more vulnerable than marginalized groups to the effects of perceiving discrimination on cardiovascular health.
• Factors other than interpersonal discrimination may more substantially contribute to racial and gender disparities in cardiovascular health.

## Introduction

Due to prominent disparities in cardiovascular outcomes between black and white women and men in the United States (Mensah & Brown, 2007; Pool, Ning, Lloyd-Jones, & Allen, 2017), researchers have examined social group-specific exposures as potential contributors to these inequities (Wyatt et al., 2003). Consistent with the dominant biomedical, individual-level orientation of epidemiological research (Krieger, 2014), the literature has largely focused on interpersonal racial discrimination as a driver of poorer cardiovascular health (CVH) within these groups (Brewer & Cooper, 2014; Ferdinand & Nasser, 2017; Krieger, 2014; Wyatt et al., 2003). Previous studies have linked reported racial discrimination to sedentary behavior, smoking, hypertension, obesity, and incident cardiovascular disease (CVD) within black and white populations (Borrell et al., 2010; Hunte & Willaims, 2009; Sims et al., 2012; Udo & Grilo, 2017; Womack et al., 2014). Because the prevalence of reported interpersonal racial discrimination is substantially higher among black persons than whites (Bey et al., under review; Krieger & Sidney, 1996; Shariff-Marco, Klassen, & Bowie, 2010; Williams, Yu, Jackson, & Anderson, 1997), these findings have generally been interpreted through the lens of differential exposure rather than vulnerability (Lewis, Williams, Tamene, & Clark, 2014). That is, a higher prevalence of disease theorized to correspond with a higher prevalence of exposure, rather than with a greater vulnerability to the effects of exposure (Brewer & Cooper, 2014; Krieger, 2014). Consequently, consensus has leaned toward an association of what has been conceptualized as “perceived” but measured as “reported” racial discrimination with the disproportionate rate of cardiovascular morbidity and mortality among blacks (Brewer & Cooper, 2014; Krieger, 2014; Wyatt et al., 2003).

Yet, while structural and interpersonal discrimination are also more prevalent among women (Author information blinde; Krieger, 2016; Kawachi, Kennedy, Gupta, & Prothrow-Stith, 1999), recent evidence showing no association of reported gender discrimination with incident CVD (Udo & Grilo, 2017), along with other recent findings inconsistent with previous evidence (Dunlay et al., 2017), calls into question unidimensional conceptualizations of discrimination as a cause of poorer CVH. A focus on differential exposure to interpersonal discrimination as underlying racial and gender disparities in CVH may prevent identification of other relevant group-specific characteristics such as varying susceptibility to the health effects of perceiving discrimination (Assari, 2018a, 2018b; Assari & Lankarani, 2015; Krieger, 2014; Peterson, Matthews, Derby, Bromberger, & Thurston, 2016; Williams et al., 2012). For example, a recent study assessing the effect of cumulative unfair treatment on subclinical CVD among a multi-ethnic sample of women found an association only among white women (Peterson et al., 2016). Such evidence supports the argument that while women and black persons are more likely to experience interpersonal gender or racial discrimination as a result of structural discrimination, men and white persons may be more susceptible to the health consequences of perceiving interpersonal discrimination (Bey et. al, under review; Bey, Waring, Jesdale, & Person, 2016; Everson-Rose et al., 2015). Whether this increased vulnerability is due to a lower tolerance for psychosocial adversity (Brown, Mitchell, & Ailshire, 2018; DiAngelo, 2011) or stress stemming from the absence of objective evidence or consensus that such experiences frequently occur to members of dominant status groups (Bey et. al, under review; Barnes et al., 2008) has yet to be determined.

Within-gender racial differences (referred to as “gendered racial” from here out) in the prevalence and severity of CVD further highlight the necessity for a stronger theoretical foundation in understanding the role of discrimination in yielding CVD disparities (Krieger, 2016). The age-adjusted likelihood of a CVD diagnosis is approximately equal for black and white men (Benjamin et al., 2017; National Quality Forum, 2017), but black women are nearly twice as likely as white women in the same age group to develop CVD (Benjamin et al., 2017; National Quality Forum, 2017). Black women are also more likely than white women or black men to develop cardiometabolic precursors to CVD (Pool et al., 2017; Robinson, Gordon-Larsen, Kaufman, Suchindran, & Stevens, 2009). Among other risk factors (Krieger, 2014; Robinson et al., 2009), researchers frequently attribute this increased risk among black women to a greater likelihood of experiencing racial and gender discrimination (Jackson, Williams, & VanderWeele, 2016; Krieger, 2016; Williams et al., 2012). Unlike the large gender disparity among whites, however, black women and men report comparable exposure to interpersonal gender and racial discrimination (Harnois & Ifatunji, 2011) even as black men develop CVD at a faster rate than black women (Pool et al., 2017; Mensah & Brown, 2007). The complex relationships of these psychosocial exposures with CVD among black and white women and men connoted in the literature point to a need for further consideration of how and in whom discrimination operates to affect risk for disease (Krieger, 2014).

**Fig. 1:**
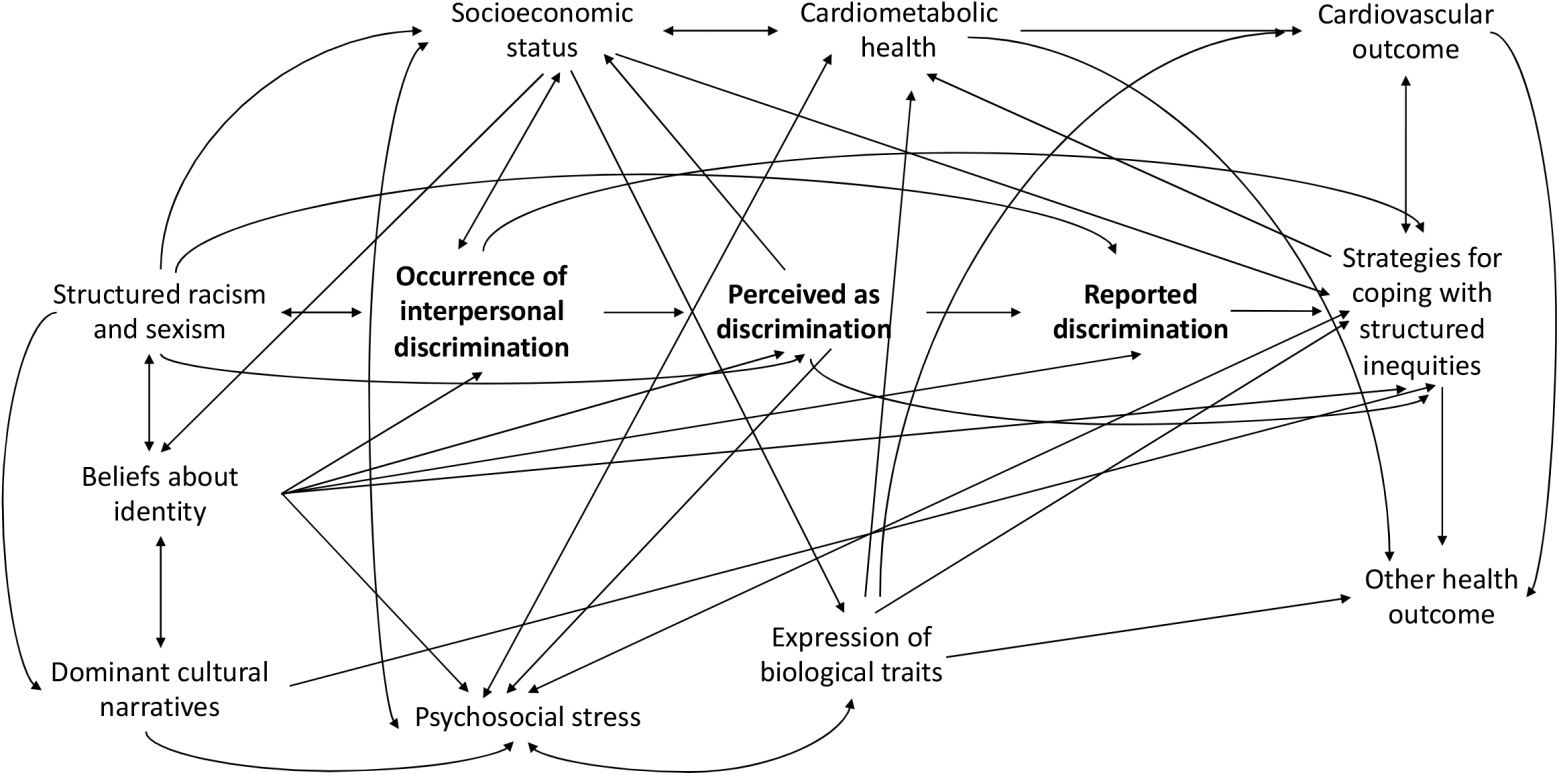
Application of the emerging Identity Pathology framework to describe potential pathways from intersecting axes of structured racism and sexism to cardiovascular disease.

Our emerging Identity Pathology framework provides a useful model for investigating these inconsistencies in the relationship of discrimination with CVD (see Fig. 1). This framework describes the health impacts of occupying multiply marginalized social positions, positing an effect of systemic race and gender inequities, as well as associated psychosocial factors, on the relationships of interpersonal discrimination with CVD. A strong body of literature within the sociological disciplines describes the racial and gender inequity inherent to the hierarchical social structure of the United States (Krieger, 2014; Kawachi et al., 1999; Williams & Mohammed, 2013; Davis, 1981; Pratto, Sidanius, & Levin, 2006). Intersectionality theory (Crenshaw, 1989, pp. 139–167), Ecosocial theory (Krieger, 2016), and the Environmental Affordances model (Mezuk et al., 2013) specifically emphasize the unique effect of multiple forms of structured inequity acting at the junction of various socially-defined characteristics to influence the distribution of health-impacting resources across dominant status and marginalized populations. Alongside these theories, evidence from the social psychological disciplines, including Social Identity (Tajfel & Turner, 1986) and Multidimensional Identity (Reynolds & Pope, 1991) theories, describe how the construction of a gendered racial identity is informed by these intersecting axes of structured oppression. Social dominance theory (Sidanius et al., 1993) further suggests that social hierarchy is supported through “legitimizing myths” or consensually shared social ideologies which position certain groups as beneficiaries of social and material resources while depriving other groups of access. Application of the Identity Pathology (IP) model to cardiovascular disease (Fig. 1) draws from these and other existing frameworks (e.g. The Jedi Public Health framework (Geronimus et al., 2016)) in explicating how observed patterns in reported interpersonal racial and gender discrimination among black and white women and men have important implications for disparities in cardiovascular disease between these groups.

The model is distinct from Intersectionality theory (Crenshaw, 1989, pp. 139–167) in that it hypothesizes the concept of identity pathology, which describes a disease-prone state characterized by certain acquired beliefs about individual or group identity that are inherently pathological, as a primary mediator of the effects of unequal social conditions on health. Constructed in the context of structured inequities such as institutional racism and sexism, these identity beliefs are informed by unique experiences at the junction of multiple social group designations and may partially account for the types of chronic diseases prevalent among different socially-defined groups. One such junction is described by the term “gendered race”. Gendered race captures the interdependent, conconmitant elements of socially-assigned gender and race categories that cannot be decomposed, neither within an individual's self-concept, nor in the mechanisms over which social inequities operate to structure privilege and marginalization based on these characteristics.

While not solely applicable to CVD, the IP model is useful for clarifying inconsistencies in the literature on interpersonal discrimination and CVD because it specifies the conditions under which—and in whom—reported experiences of interpersonal discrimination will be measured as damaging to CVH and lead to the development of disease. The major premise of the IP framework is that in moderating whether and how exposure to chronic psychosocial stressors will affect disease, socially-constructed identities can be rendered pathological. Gaining a more thorough understanding of the effects of psychosocial stressors on disease outcomes therefore requires additional clarity on the ways in which identity shapes the experience of stress.

As applied to CVD disparities and interpersonal discrimination, the model makes three central assertations. First, that in order to more accurately capture the effects of interpersonal discrimination on cardiovascular health and health disparities, multiple aspects of the discrimination experience must be considered in the design, analysis, and interpretation of health-related studies. Secondly, the IP framework posits that experiences of interpersonal discrimination are fundamentally based in historically-structured inequities that impact on each dimension of the discrimination process in health-relevant ways. Finally, the model purports that the precision with which reported experiences map onto perceptions and intentionally or implicitly-driven acts of discrimination depend on a variety of psychosocial characteristics, one of the most important of which is an individual's beliefs about their gendered racial identity. Importantly, the framework does not assert that compounded inequity necessarily translates to greater likelihood of a specific disease outcome. Instead, the framework argues that the lived experience of race and gender in a society which advantages some groups while disadvantaging others (Krieger, 2016; Williams et al., 2012) based on these identities yields variation in the efficacy of health-protective factors. This variation in turn manifests as a differential vulnerability to disease across gendered race groups (Bey et al., 2016, 2018a; Crenshaw, 1989, pp. 139–167; Krieger, 2014).

Extant epidemiological literature in accordance with the IP framework has identified gendered racial differences among black and white women and men in lung cancer treatment and mortality (Williams et al., 2012); in the protective effects of income on depression (Assari, 2018a, Assari, 2018b); in the association of depression with mortality (Assari, 2018a, Assari, 2018b); in the association of discrimination with CVD risk factors (Borrell, Diez-Roux, Kiefe, Williams, & Gordon-Larsen, 2013); and in the link between chronic stress and depression (Bey et al., 2016, 2018b); among other exposure-health combinations. These studies suggest that the contribution of discrimination to disparities in CVH may extend beyond gendered racial variation in exposure to gendered racial differences in the effect of perceiving interpersonal discrimination.

The IP model argues that this variability in effect across gendered race groups can be attributed to differing manifestations of identity pathology. Due to the relationship between identity pathology and the experience of interpersonal discrimination, the experience being captured in reported discrimination among different gendered race groups must necessarily be different. For men reporting frequent experiences of gender discrimination, these experiences are less likely to reflect objective encounters with discrimination as traditionally conceptualized and are more likely to signify that these men feel they are being deprived of the entitlements they believe they are due as a result of their manhood. Similarly, reporting of multiple encounters with racial discrimination by white persons likely indicates encounters in which these individuals believe they were deprived of entitlements due to them as white persons. Regardless of the accuracy of their reporting, the perception of what members of dominant status groups consider discrimination can be stressful enough to have a measurable impact on their cardiovascular health. This effect may be exacerbated by their recognizing the inconsistencies of their perceptions with the way that society defines experiences of discrimination.

Moreover, even among those whom the occurrence, perception, and reporting of discrimination overlap with high accuracy, differences in beliefs about the significance of being perceived and treated as inferior by another group will influence the stressfulness of perceiving discrimination. Finally, identity beliefs associated with gendered race also shape how individuals will cope with the reality of being perceived and treated as inferior, thereby creating another source of variability in the effect of reported interpersonal discrimination on CVH. Because increased exposure to social stressors among marginalized groups may yield an array of adaptive coping strategies that are protective against the health consequences of psychosocial adversity, the IP model predicts, perhaps counterintuitively, that the association between reports of racial and gender discrimination and declining CVH will be stronger among members of dominant status groups.

Exploring how CVH is impacted by the distinct social group characteristics to which individuals attribute experiences of discrimination allows us to test hypotheses generated from the IP framework. Specifically, our analysis evaluated whether the associations of simultaneously reported interpersonal experiences of racial and gender discrimination, compared with racial or gender discrimination alone, or no discrimination, with cardiovascular health 23 years later among a community-based sample of black and white women and men in four U.S. cities was stronger among white men than other gendered race groups due to the hypothesized associations of reported interpersonal discrimination and identity pathology in this group.

## Methods

### Study design and participants

The Coronary Artery Risk Development in Young Adults (CARDIA) study is an ongoing community-based prospective cohort study of risk factors for cardiovascular disease conducted in four U.S. centers (Birmingham, AL; Minneapolis, MN; Chicago, IL; and Oakland, CA). 5114 self-reported black and white persons, aged 18–30 years at baseline examination (1985–1986), were recruited primarily from random-digit dialing of community lists and random selection from a health-care plan (Cutter et al., 1991; Friedman et al., 1988). The goal of recruitment was to balance on gender and race; participants aged 18–25 years and those older than 25; and those attaining a high school education or less, and those with more education, across the four centers. The institutional review board at each center approved the CARDIA study protocol and informed consent was obtained from each participant. Following the initial examination, participants were re-surveyed at years 2, 5, 7, 10, 15, 20, 25, and 30 post-baseline.

### Reported discrimination

The primary exposure was reported interpersonal discrimination based on race and gender as a source of chronic, toxic psychosocial stress. Discrimination was first assessed in the CARDIA study at 7 years post-baseline, using the valid and reliable Experiences of Discrimination scale (Krieger & Sidney, 1996). Participants reported having ever experienced discrimination, been prevented from doing something, or been hassled or made to feel inferior (yes/no) in any of the following settings: at school; getting a job; at work; at home; getting medical care; getting housing; by the police or courts; or on the street or in a public setting. At year 7, the racial discrimination scale excludes “at home” and the gender discrimination scale excludes “getting housing” or “by the police or courts”.

Previous CARDIA studies have treated discrimination as a 4-category variable to capture the extent and persistence of discrimination in only one subscale (race or color) across years 7 and 15 (Borrell et al., 2013). Rather than conceptualizing the combined reporting of racial and gender discrimination as an indicator of a greater degree of exposure, we consider simultaneously reported racial and gender discrimination as a measure of the way in which individuals interpret their experience of structural inequity. Because preliminary analyses of CARDIA data show that the prevalence of reported race and gender discrimination is comparable at years 7, 15, and 25 within each gendered race group, we used discrimination reported at year 7 only. Each type of discrimination (based on gender or race or color) was categorized as reported in 0, 1, or ≥ 2 domains, as these categories are shown to represent variable health risk (Borrell et al., 2013). To contrast single with multiple forms of discrimination, the main exposure variable included five categories: none (no racial or gender discrimination reported); any racial only (only racial discrimination in one or more settings); any gender discrimination (only gender discrimination in one or more settings); any racial and gender discrimination, in <2 settings; and racial and gender discrimination, in ≥2 settings. Information on gendered race (black women/black men/white women/white men) and city (as measured by study center) was taken from data collected at baseline. Less than 5% reported geographic migration between baseline and year 7, so the study center in which the last exam (prior to the first reported experience of discrimination) was conducted was used for geographic location.

### Cardiovascular health score

The primary outcome was a cardiovascular health score, based on the American Heart Association (AHA) Life's Simple 7 Cardiovascular Health scoring (Pool et al., 2017). Because no dietary measures were included in the analysis, the composite score comprised six rather than seven different measures including two behavioral factors (smoking status and physical activity) and four cardiometabolic factors (hypercholesterolemia, hypertension, obesity, and diabetes defined per AHA and National Heart, Lung, and Blood Institute guidelines). The total score was calculated as a summation of points assigned for each factor. Smoking status was operationalized as self-report of never (2 points), former (1 point), or current (0 points). Physical activity was defined by CDC guidelines for promoting cardiovascular health (ref) as ≥75 min/week vigorous physical activity (VPA) or ≥150 min/week of moderate physical activity (MPA) (2 points); <75,>0 min/week VPA or <150,>0 min/week MPA) (1 point); and none (0 points). Hypercholesterolemia was operationalized as total cholesterol <200 mg/dL (2 points); 200–239 mg/dL (1 point); and ≥240 (0 points); hypertension as the average of three systolic/diastolic readings <120/80 (2 points); 120/81–139/89 (1 point); and ≥140/90 (0 points); obesity as body mass index (BMI) of <25 kg/m^2^ (2 points); 25–29.9 (1 point); ≥30 (0 points); and diabetes as fasting blood glucose of <100 mg/dL (2 points); 100–125.9 (1 point); and ≥126 (0 points). Total scores ranged from 0 to 12, with higher points indicating healthier status. The cardiovascular health score was treated as a continuous variable.

### Gendered race

CARDIA measures only binary biological sex and self-reported race; we use a race-by-gender variable to operationalize gendered race.

### Covariates

Potential confounders for this analysis are limited to age (continuous), and study center. Other potentially relevant sociodemographic variables such as annual family income, marital status, and education level were not included as confounders because they were conceptualized as potential mediators. However, given hypothesized racial differences in the pathways from interpersonal discrimination to CVD, we also conducted sensitivity analyses with models additionally adjusting for SES (as measured by years of education) among white women and men.

### Statistical analysis

Descriptive statistics including age, study center, type and level of discrimination, and CVH scores were calculated for each gendered race group using Pearson's chi-square test for categorial variables and t-tests for continuous variables. Multivariable linear regressions were used to evaluate the associations between category of reported discrimination at year 7 and CVH scores at year 30 (or the last follow-up) for those reporting both racial discrimination and gender discrimination (either in <2 or ≥2 settings) compared with those reporting racial or gender discrimination alone, or no discrimination, stratified by gendered race group. To address potential bias from attrition and gendered race group compositional effects, we also conducted sensitivity analyses weighting effect estimates. Weights were calculated using combined inverse probability of missing at year 30 (age, study center, and years of education) and group propensity score matching (age, study center, and years of education) for each gendered race group relative to white men. Analyses were conducted using Stata 14.0 (StataCorp, Texas).

## Results

Exclusion of those missing data on race or gender discrimination at year 7, CVH score, or included covariates resulted in an analytic sample of 3758 participants. There was considerable variation in the prevalence of each level and type of discrimination reported by each gendered race group, and in CVH scores across categories of discrimination (Table 1). Among black women and men, 84% and 88%, respectively, reported some form of racial or gender discrimination exposure, compared with 67% of white women and 42% of white men. Of these, the proportion of black women (48%) and men (42%) reporting both racial and gender discrimination in ≥2 settings was comparable, while twice as many white women (10%) as white men (5%) reported exposure to both forms of discrimination. Within each gendered race group, unadjusted CVH scores also varied by level and type of discrimination. Among black women and white men, those reporting only racial discrimination had the highest CVH scores of their groups, while white women reporting only racial discrimination had lower scores than white women reporting in all other categories. For men of both races, those reporting both types of discrimination in ≥2 settings had lower CVH scores than those in their groups reporting no exposure. For women, those reporting dual exposure had approximately the same scores as women reporting no experiences of discrimination.

**Table 1:** Reported Racial and/or Gender Discrimination and Cardiovascular Health Score (CVH) by Gendered Race: CARDIA, 1992–2016.

	Black women		Black men		White women		White men	
N	1039		743		1045		931	
Yr 30 age, mean yrs (SD)	54.6 (3.8)		54.3 (3.7)		55.6 (3.4)		55.5 (3.3)	
Reported Discrimination	Yr 7 Disc., %	Yr 30 CVH Score^a^, mean (SD)	Yr 7 Disc., %	Yr 30 CVH Score^a^, mean (SD)	Yr 7 Disc., %	Yr 30 CVH Score^a^, mean (SD)	Yr 7 Disc., %	Yr 30 CVH Score^a^, mean (SD)
None	15.7	7.6 (2.0)	12.4	8.3 (1.5)	22.6	8.7 (2.1)	57.8	8.7 (1.8)
Only racial	12.1	8.0 (1.8)	27.5	8.2 (1.8)	2.0	8.5 (2.0)	13.0	9.2 (1.6)
Only gender	6.8	7.3 (2.0)	3.1	8.5 (2.1)	47.0	9.0 (2.0)	13.6	8.7 (1.9)
Any racial or gender, in <2 settings	17.5	7.7 (1.8)	15.1	8.3 (1.9)	18.0	9.0 (2.0)	10.8	8.5 (2.0)
Both racial and gender, in ≥2 settings	47.7	7.8 (1.9)	41.9	8.0 (1.7)	10.4	8.8 (2.0)	4.8	8.2 (1.8)

a Cardiovascular Health scores are calculated based on data collected in year 30 or the last follow-up, using six components with a total possible 12 points: body mass index, total cholesterol, systolic blood pressure, fasting glucose, smoking status, and physical activity. Higher scores indicate better health.

Adjusted differences in CVH score at year 30 across levels and type of discrimination for each gendered race group can be found in Table 2. Among black men, neither racial nor gender discrimination, alone or in combination, was statistically significantly associated with CVH score. For black women, those reporting racial but not gender discrimination had higher CVH scores compared with black women who reported no discrimination (ß = 0.4, 95% CI*:* 0.0, 0.8). White women who reported experiencing only gender discrimination likewise had a higher score (ß = 0.3, 95% CI: 0.0, 0.6) compared white women reporting no discrimination. Among white men, whether the CVH score difference was positive or negative depended on both the type and level of discrimination. White men reporting only racial discrimination saw a positive difference of 0.4 (95% CI: 0.1, 0.8) compared with white men reporting no discrimination, while those reporting racial and gender discrimination in ≥2 settings had lower CVH scores (ß = −0.6, 95% CI: 1.1, −0.1).

**Table 2:** Adjusted Difference in Cardiovascular Health Score^a^ for Categories of Reported Racial and/or Gender Discrimination by Gendered Race^b^: CARDIA, 1992–2016.

	Black women	Black men	White women	White men
Discrimination (year 7)	ß (95% CI)	ß (95% CI)	ß (95% CI)	ß (95% CI)
None	ref	ref	ref	ref
Any racial only	**0.4 (0.0, 0.8)**	−0.1 (−0.5, 0.4)	−0.3 (−1.2, 0.6)	**0.4 (0.1, 0.8)**
Any gender only	−0.3 (−0.8, 0.2)	0.2 (−0.6, 1.0)	**0.3 (0.0, 0.6)**	0.0 (−0.4, 0.3)
Any racial or gender, in <2 settings	0.1 (−0.3, 0.5)	0.0 (−0.5, 0.5)	0.2 (−0.2, 0.6)	−0.2 (−0.6, 0.1)
Both racial and gender, in ≥2 settings	0.2 (−0.1, 0.6)	−0.3 (−0.7, 0.1)	0.0 (−0.4, 0.4)	**−0.6 (-1.1, -0.1)**

Bolded values are statistically significant at p < 0.05.

a Cardiovascular health scores are calculated based on data collected in year 30 or the last follow-up using six components: body mass index, total cholesterol, systolic blood pressure, fasting glucose, smoking status, and physical activity. Higher scores indicate better health.

b Models are adjusted for age and geographic location.

In the sensitivity analyses (data not shown), which included models additionally adjusting for years of education in white women and men, all effect estimates became non-significant, except among white men reporting only racial discrimination, among whom the coefficient was ß = 0.4 (95% CI 0.1, 0.7).

## Discussion

Our findings identified important characteristics of the relationships between reported racial and gender discrimination and cardiovascular health (CVH). Black women and men were comparable in likelihood of reporting experiences of racial and gender discrimination in multiple settings, while twice as many white women as men reported experiencing both types of discrimination. In addition to gendered race differences in magnitude of exposure, there were differences in the associations between reported gender and racial discrimination and CVH, suggesting differential vulnerability. Black women reporting only racial discrimination had better CVH on average than black women who reported none. No statistically significant associations were found among black men. Among white women, reporting any gender discrimination predicted higher CVH scores than reporting no discrimination. For white men, predicted CVH scores were higher for those reporting any racial discrimination, and lower for those reporting racial and gender discrimination in at least two settings, than in those reporting no discrimination. While the differences in CVH scores may appear small, a 1-point difference in CVH score could signify the difference between, for example, controlled and uncontrolled hypertension, or between meeting clinical criteria for diabetes and not having diabetes. Therefore, even a 0.5 decrease in CVH score represents significant deterioration in cardiovascular health.

These results are consistent with the body of evidence describing varied experiences of interpersonal racial and gender discrimination among black and white women and men. Previous studies using CARDIA data (Bey et al., under review; Krieger & Sidney, 1996; Borrell et al., 2013) as well as other community-based samples (Borrell et al., 2010; Hunte & Willaims, 2009) have shown the prevalence of reported racial discrimination to be slightly higher among black men than women and similar between white women and men. Also consonant with our findings, other studies show a higher prevalence of reported gender discrimination among women than men, but only among white persons; black men have previously been shown to report levels of exposure to gender discrimination comparable to black women (Bey et al., under review; Jackson et al., 2016).

On the other hand, our findings contrast with those describing a link between racial discrimination and poorer cardiovascular health among black persons (Borrell et al., 2013; Brewer & Cooper, 2014; Krieger, 2014). Though inconsistent, the literature has demonstrated associations of reported racial discrimination with CVD risk factors including diet, hypertension, smoking, sedentary behavior, obesity, and inflammation (Borrell et al., 2010, 2013; Cunningham et al., 2012a, 2012b; Womack et al., 2014), as well as social predictors of CVD such as marital status, socioeconomic position, and education, in both black women and men (Krieger, 2014; Murry, Brown, Brody, Cutrona, & Simons, 2001; Williams et al., 1997). In this study, we did not find a statistically significant association between racial discrimination and poorer CVH within these groups. Other cross-sectional analyses (Albert et al., 2010; Hunte & Willaims, 2009) and the only study prospectively examining the relationships of racial discrimination with incident CVD exclusively among black women and men have also failed to find a connection (Dunlay et al., 2017). Taken together, these findings offer evidence that traditionally accepted risk factors may be poorer predictors of CVD among black persons, as has been previously posed (Bey et. al, under review). Accordingly, while interpersonal racial discrimination may increase the likelihood that black women and men develop cardiometabolic risk factors for CVD, other factors integral to the experience of multiply marginalized identities may have a much more substantial impact on the development of CVD in these groups. As these other potential risk factors remain understudied (Krieger, 2014, 2016), the long history of investigating interpersonal discrimination as a cause of poorer health has done little to expand an understanding of CVD disparities between black and white women and men. Consequently, there is limited evidence that a continued focus on interpersonal discrimination as a cause of increased CVD burden among black women and men is even warranted.

In addition to suggesting alternative causes of higher CVD morbidity and mortality among marginalized groups, our emerging IP model theorizes that discrepancies between the occurrence, perception, and reporting of interpersonal discrimination contribute to the observed varibility in the associations of reported racial and gender discrimination with CVH among black and white women and men (see Fig. 1). The model suggests that for some gendered race groups in certain places and settings, reported discrimination is more likely to reflect interactions that meet objective standards of inequitable treatment. In these cases, acknowledging experiences that actually occur may be beneficial for health, while denying may lead to increased stress and stress-related pathology regardless of one's gendered race group (Brenner, Diez-Roux, Gabreab, Schulz, & Sims, 2018; Cunningham et al., 2012a, 2012b). From building social networks based on shared experiences to enabling the development of healthier coping behaviors (Borrell et al., 2013; Chae, Lincold, & Jackson, 2011), recognizing and acknowledging the discrimination one encounters may allow for chronic stress relief that reduces risk for CVD associated with discrimination exposure (Brenner et al., 2018; Cunningham et al., 2012a, 2012b). Reported experiences of racial and gender discrimination may thus be meausured as protective among those against whom such experiences actually occur.

This would explain why, relative to those of their gendered race group reporting no discrimination, white women reporting exposure to only gender discrimination on average had higher CVH scores. Simarly for black women reporting only racial discrimination; the higher CVH score among this group of black women is consistent with the theory that reporting experiences of discrmination that actually occur may indicate a tendency for health-promoting coping strategies. Importantly, the difference in the direction of effect between black women who acknowledge only experiences of racial discrimination and those acknowledging only gender discrimination suggests a distinction between these groups—which may be true of white women as well. The higher CVH scores among black women claiming to only have experienced racial discrimination in their lifetime suggests that among black women there is a subgroup of individuals whose racial identities predominate their self-concepts and who are therefore more likely to attribute experiences of discrimination they encounter to their race (Chae et al., 2011). Rather than indicating a denial of gender-based discrimination, this pattern may instead represent a difference in attribution. Black women reporting *only* gender discrimination may on the other hand actually be denying the racial discrimination directed against them, which in turn may lead to (or be indicative of) coping strategies that accelerate deterioration in CVH. In this way, regardless of attribution, acknowledging everyday experiences of discrimination that actually occur may in fact be protective of CVH, while denying these experiences may be detrimental, as is indicated by previous cross-sesctional research (Brenner et al., 2018; Chae et al, 2010, 2011) and a recent longitudinal study (Dunlay et al., 2017).

To fully account for our results in the context of this theory, it is important to note that across the four gendered race groups, reporting or not reporting exposure likely signify different health-relevant psychological and emotional states (Bey et al., under review; Chae et al., 2011; Chae et al., 2010). The relatively low percentage of black women who reported experiencing no racial or gender discrimination did so despite a considerable body of evidence to the contrary, indicating a measure of denial or “tough it out” mentality in this group (Chae et al., 2011) distinct from the evidence-based reasons that a much greater proportion of white men would report no exposure. Even within gendered race groups, the meaning of reported exposure to discrimination may vary. As proposed by the IP framework, (Bey et. al, under review) white men reporting few experiences of racial discrimination may subscribe to identity paradigms distinct from those in their group reporting both racial and gender discrimination in multiple settings. The framework posits that among white persons, reported experiences of racial discrimination in only one setting (e.g. at school) may be more likely to meet objective standards of discriminatory treatment, particularly in metropolitan areas such as Oakland with a greater degree of racial integration. Accordingly, higher average CVH scores among white men who reported only racial discrimination would not be inconsistent with a protective effect of reporting interpersonal experiences of discrimination that meet objective measures. That is, white men who reported only exposure to racial discrimination were likely the white men for whom the overlap of the occurrence, perception, and reporting of discrimination was relatively accurate. As the IP model predicts, in such cases, there is likelihood that reported discrimination will be measured as protective of CVH. That the positive effect on CVH among white men reporting only racial discrimination persisted even after adjusting for SES further supports this assertation.

Among white persons in other places and settings, perceiving discrimination in the absence of external validation of such experiences may represent endorsement of belief systems which generate chronic, toxic stress in a society proclaiming ideals contrary to these beliefs—beliefs about identity which the IP framework positions as pathological. In support of this theory, one study examing reported experiences of racial discrimination and inflammation found the association to be highest among white women reporting exposure in at least three settings while no associations were found among black women or men reporting in as many settings (Cunningham et al., 2012a, 2012b). Further, having had fewer opportunities than black persons to become accustomed to the psychological hardship of perceiving unequal treatment (Brown et al., 2018; DiAngelo, 2011), white persons may be more vulnerable to the negative effects of perceiving racial and gender discrimination on CVH (Brown et al., 2018; Cunningham et al., 2012a, 2012b; DiAngelo, 2011).

### Limitations

There are some limitations to this analysis requiring acknowledgment. CVH scores are taken from data at the last follow-up. For some participants, this is as early as year 15. Because CVH scores are associated with age, there is potential that those participants retained through year 30 have lower CVH scores because they are older. However, we do not believe differential dropout rates significantly impacted on effect estimates due to the high (and similar) proportion of participants in each gendered race group whose CVH scores were calculated based on year 30 data, in addition to including age of participants in regression models. Further, rate of attrition through year 30 in CARDIA is highest among black men and lowest among white women, but not statistically different between black women and white men. While the prevalence of simultaneously reported racial and gender discrimination was slightly higher among white men dropouts than retained white men (6% vs. 4%), the exposure was lower among black women dropouts (30%) than those who remained in the study at year 30 (40%). On the other hand, CVD incidence among black women and white men who dropped out by year 30 were higher than their retained counterparts but not statistically different from one another. So, while attrition may be differentially associated with the exposure, it is unlikely to be differentially associated with the outcome across these groups. In fact, patterns among dropouts reinforce our results. We take these data as evidence that it is unlikely our findings can be attributed to compositional effects.

CVH scores for this study were modified from the AHA's Life's simple 7 and do not include dietary measures. For this reason, CVH scores may not reflect cardiovascular health with the same accuracy and cannot be compared directly to other studies using this measure. Despite this modification, we believe that the included markers sufficiently represent risk for poorer cardiovascular health as each condition included in the composite score has been previously shown to individually correlate highly with poor cardiovascular outcomes. Therefore, we believe that the modification does not negate the validity of our findings.

The sensitivity analyses we conducted revealed that adjusting for years of education attentuated effect estimates among white women who reported only gender discrimination and white men who reported both racial and gender discrimination. Because SES is known to highly correlate with reports of racial discrimination and with CVD among whites, these results may indicate a spurious association of CVH with reported discrimination in this study. However, as noted, the effect estimates among white men who reported only racial discrimination persisted despite accounting for SES. This suggests that rather than contradicting the IP theory, the additionally adjusted analyses offer some evidence that white men who report experiencing only racial discrimination may be fundamentally distinct from those reporting both racial and gender discrimination in multiple settings. The distinctions between these groups of white men, potentially regarding identity beliefs about racial and gender hieararchy that influence perceptions of discrimination, also appear to have important implications for CVH. Additional research is needed to empirically assess whether identity pathology accounts for any of the increased risk for CVD prevalent among white persons of lower SES.

## Conclusions

This study offers evidence of important variation in the health effects associated with reported racial and gender discrimination among black and white women and men, while also providing empirical support for the emerging IP framework (Bey et. al, under review). Our findings suggest that the literature remains conflicted on the relationships of interpersonal discrimination with CVH perhaps because the associations vary between these groups in direction and magnitude, in ways that cannot be account for with simple adjustment for confounding. Black persons and women may be at greater risk for exposure, but white men appear to be most susceptible to the negative effects of perceiving multiple forms of discrimination on CVH. Further, within-race gender differences indicate racial heterogeneity in effects that also should not be overlooked. These results highlight the necessity for additional research in a number of areas. Studies with larger sample sizes can statistically verify differences in the effect estimates between these groups and allow for a more confident interpretation of findings. As previously postulated (Krieger & Sidney, 1996), the experience of interpersonal discrimination among white persons appears fundamentally distinct from that of black persons in ways that impact on health and disease. Qualitative methods are necessary to explore the meaning and health significance of reported discrimination in more depth within white populations, in part to clarify which other psychosocial factors are actually being captured in reported experiences of discrimination.

In trying to understand the factors driving increased CVD burden among black women and men, more attention should also be given to other characteristics comprising the unique social experiences of these groups. In a society still fraught with structured racial and gender inequity, multiply marginalized individuals may be forced to navigate in ways that more substantially contribute to their increased risk for disease than being mistreated on a personal basis. If the intent of examining interpersonal discrimination as a predictor of health is to identify possible interventions on CVD disparities, future research should consider more in-depth exploration of the causes behind differential reporting of discrimination, and whether these predecessors are better predictors of CVD. Such lines of investigation may yield more comprehensive explanations of persistent CVD inequities and identify targets for intervention more amenable to change.

## Declaration of interest

The authors declare no conflicts of interest.

## Ethics statement

Approval for the use of Human Subjects data in the CARDIA study was granted by the Institutional Review Boards of the following institutions:

University of Alabama at Birmingham

Northwestern University

University of Minnesota

Kaiser Permanente, Research Division.
