# Supplementary material for: Intersectional effects of racial and gender discrimination on cardiovascular health vary among black and white women and men in the CARDIA study
Source: SSM Popul Health. 2019 Jul 4;8:100446. doi: 10.1016/j.ssmph.2019.100446 (PMC6620618; doi:10.1016/j.ssmph.2019.100446)
Supplement: Multimedia component 1 [file mmc1.docx]

**Abstract**

**Supplementary Table 1.** Weighted Difference in Cardiovascular Health Score^a^ for Categories of

Reported Racial and/or Gender Discrimination by Gendered Race^b^: CARDIA, 1992-2016

|  | Black women | Black men | White women | White men |
| --- | --- | --- | --- | --- |
| Discrimination (year 7) | ß (95% CI)  ref  0.4 (0.0, 0.8)  -0.3 (-0.8, 0.2)  0.1 (-0.3, 0.5)  0.2 (-0.1, 0.6) | ß (95% CI)  ref  -0.2 (-0.6, 0.2)  0.2 (-0.6, 1.0)  0.0 (-0.5, 0.5)  **-0.4 (-0.8, 0.0)** | ß (95% CI)  ref  -0.2 (-1.1, 0.7)  **0.4 (0.0, 0.7)**  0.3 (-0.2, 0.7)  0.1 (-0.4, 0.5) | ß (95% CI)  ref  **0.4 (0.1, 0.8)**  0.0 (-0.4, 0.3)  -0.2 (-0.6, 0.1)  **-0.6 (-1.1, -0.1)** |
| None |  |  |  |  |
| Any racial only |  |  |  |  |
| Any gender only |  |  |  |  |
| Any racial or gender, in <2 settings |  |  |  |  |
| Both racial and gender, in ≥2 settings |  |  |  |  |

^a^Cardiovascular health scores are calculated based on data collected in year 30 or the last follow-up using six components: body mass index, total

cholesterol, systolic blood pressure, fasting glucose, smoking status, and physical activity. Higher scores indicate better health.

^b^Models are adjusted for age and geographic location.

Bolded values are statistically significant at p <0.05.

^d^Weights were calculated using combined inverse probability of missing at year 30 (age, study center, and years of education) and group propensity

score matching (age, study center, and years of education) for each gendered race group relative to white men.
